# ATP Bioluminometers Analysis on the Surfaces of Removable Orthodontic Aligners after the Use of Different Cleaning Methods

**DOI:** 10.1155/2016/5926941

**Published:** 2016-05-08

**Authors:** Luca Levrini, Alessandro Mangano, Silvia Margherini, Camilla Tenconi, Davide Vigetti, Raffaele Muollo, Gian Marco Abbate

**Affiliations:** ^1^Department of Surgical and Morphological Sciences, Oro Cranio Facial Disease and Medicine Research Center, Dental Hygiene School, University of Insubria, Via Giuseppe Piatti 10, 21100 Varese, Italy; ^2^Oro Cranio Facial Disease and Medicine Research Center, University of Insubria, 21100 Varese, Italy; ^3^Department of Surgical and Morphological Sciences, University of Insubria, 21100 Varese, Italy

## Abstract

*Purpose*. The aim was to quantify the bacteria concentration on the surface of orthodontic clear aligners using three different cleaning methods. Furthermore the objective was to validate the efficacy of the bioluminometer in assessing the bacteria concentration.* Materials and Methods*. Twenty subjects (six males and fourteen females) undergoing orthodontic therapy with clear aligners (Invisalign® Align Technology, Santa Clara, California) were enrolled in this study. The observation time was of six weeks. The patients were instructed to use different cleaning methods (water, brushing with toothpaste, and brushing with toothpaste and use of sodium carbonate and sulphate tablet). At the end of each phase a microbiological analysis was performed using the bioluminometer.* Results*. The highest bacteria concentration was found on aligners cleaned using only water (583 relative light units); a value of 189 relative light units was found on aligners cleaned with brushing and toothpaste. The lowest bacteria concentration was recorded on aligners cleaned with brushing and toothpaste and the use of sodium carbonate and sulfate tablet.* Conclusions*. The mechanical removal of the bacterial biofilm proved to be effective with brushing and toothpaste. The best results in terms of bacteria concentration were achieved adding the use of sodium carbonate and sulfate tablet.

## 1. Introduction

Traditional fixed orthodontic appliances lead to a change in the quantity and in the composition of oral microbiota. Fixed orthodontic devices cause plaque accumulation, impede correct professional hygiene procedures, and potentially cause enamel demineralization, tooth decay, and periodontal disease [1–6]. Digital dentistry is a fast moving field and new technologies give both the clinicians and patients new treatment possibilities. In 1999 a new orthodontic appliance based on a polymer composed of a chain of organic units joined with urethane links was introduced (Invisalign, Align Technology, Santa Clara, California) and produced with a CAM (computer aided manufacturing) technology as a removable appliance able to gradually move the teeth according to a computer designed treatment plan. The introduction of this technology gave the patients the possibility to better control the oral hygiene. In fact, the use of removable orthodontic devices guarantees a normal professional hygiene cleaning, thus reducing the risk of developing plaque related diseases [7–9]. The use of removable clear aligners showed, also, a better patient compliance in terms of oral hygiene procedures [10]. In the case of removable aligners it is important that before use they are cleaned and without bacteria. A correct hygiene is able to impede the accumulation of bacteria on the surfaces, thus avoiding the potential risk of spreading bacteria on teeth surfaces and periodontium. Therefore, it is important to clean and disinfect the removable aligners, but information given to patients is often incomplete and unclear. This could be attributable to a lack of evidence in the scientific literature; the same problem could be related also to other removable orthodontic appliances [11, 12]. The aim of this study was to evaluate the efficacy in removing the bacterial biofilm on clear aligners using three different cleaning methods. Furthermore the reliability of bioluminometer was tested.

## 2. Materials and Methods

### 2.1. Patient Population

Twenty (6 males and 14 females) consecutive patients undergoing orthodontic treatment with clear aligners (Invisalign, Align Technology, Santa Clara, California) referring to the Department of Orthodontics of the University of Insubria with age ranging from 18 to 30 years were enrolled in this study. All patients were informed of the nature of the study to be carried out on an individual basis and read and signed a written consent form. The study protocol was conducted in accordance with the Helsinki Declaration of 1975, as revised in 2007. The study protocol was approved by the Ospedale di Circolo e Fondazione Macchi Ethics Committee, Varese.

### 2.2. Inclusion and Exclusion Criteria

Inclusion criteria were as follows: Class I skeletal relationship, normodivergent Frankfort mandibular-plane angle, age > 18, and no active periodontal disease.

Exclusion criteria were as follows: smoking habit, presence of fixed bridges/crowns or partial dentures, previous periodontal nonsurgical treatment (such as full-mouth disinfection, quadrant-by-quadrant therapy, and full-mouth debridement) within the past year, and medications such as antibiotics, steroids, or nonsteroidal anti-inflammatory drugs within the past 6 months.

### 2.3. Study Design, Evaluation of Total Biofilm, and Statistical Analysis

Before taking part in the study all subjects were motivated and instructed to a correct oral hygiene by one operator (CT). All patients were instructed to use a manual toothbrush with a rolling technique. To reduce bias patients were provided with the same oral hygiene products (anticaries toothpaste, mouthrinse, and interdental floss). All subjects underwent professional dental cleaning by one operator (CT) before the study period. Each patient received three series of aligners, each to be worn for 2 weeks, and was asked to use different cleaning procedures over the 6 weeks of their application. At the end of each two-week stage a microbiological sample was obtained from the aligners by means of sterile swab. The patients were asked to clean the clear aligners using three different cleaning methods described as follows:

 T1 (water—W): during the first two weeks patients were asked to remove the clear aligners before eating and to rinse the aligners in cold running water for 15 seconds.
 T2 (toothbrush—TB): for the second two weeks before eating patients were asked to remove the aligners and to brush them for at least 30 seconds with a soft toothbrush and toothpaste with a relative dentin abrasion value of less than 100.
 T3 (tablet and toothbrush—TBT): all the subjects were asked to clean their appliances daily for at least 20 minutes by soaking them in cold water in which effervescent tablets containing sodium carbonate and sulfate (Invisalign Cleaning System, Align Technology, San Jose, CA, USA) had been dissolved. Before wearing the aligners, the patients were also instructed to brush them for at least 30 seconds with a soft toothbrush and toothpaste with a relative dentin abrasion value of less than 100. At the end of each 2-week stage, bioluminometer analysis was carried out. A Wilcoxon match paired test was used. The level of significance was set at 0.05. All statistical analyses were run on the MedCalc® software (MedCalc Software bvba, Ostend, Belgium).

### 2.4. Bioluminometer Validation

A crossed analysis was carried out in order to evaluate the reliability of the bioluminometer values. A microbiological sample was obtained and analysed. The total biofilm value was evaluated using two different methods. A sample of saliva was obtained for both analyses. The traditional LB Agar culture was carried out counting the CFU (colony-forming unit). The bioluminometer gives a bacteria concentration value expressed in RLU (relative light units). A comparison of the values obtained with the two different methods was done.

### 2.5. Bioluminometer Microbiological Analysis

A microbiological analysis was carried out using the Bioluminometer System Sure II Plus (RG Strumenti, Parma, Italy) with the SuperSnap kit (RG Strumenti, Parma, Italy) according to the manufacturer's instructions. The sample was collected passing the SuperSnap kit on the aligners from molar to molar; a round movement was performed on the molars while a simple scraping was performed on the other parts of the aligners. The samples were then stored in a solution for the bacterial lysis and for the chemiluminescence. The sample was stored for 4 hours at 4°C before proceeding with the chemiluminescence analysis.

## 3. Results

### 3.1. Bioluminometer Validation

**Figure 1:**
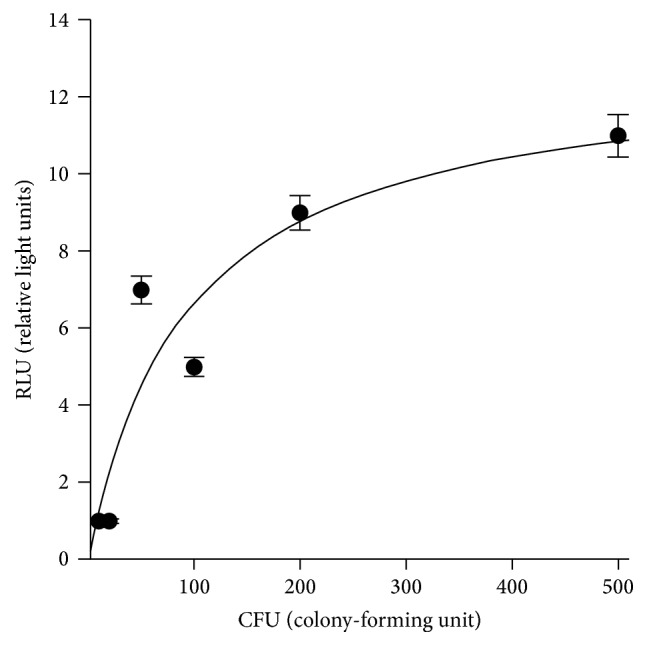
Linear relationship graph.

A correlation was found between the results obtained with the Bioluminometer and the LB Agar culture. A proportional relationship was found between UFC and the RLU values. A linear relationship was found until 200 UFC value (Figure 1).

### 3.2. Bioluminometer Microbiological Analysis

**Figure 2:**
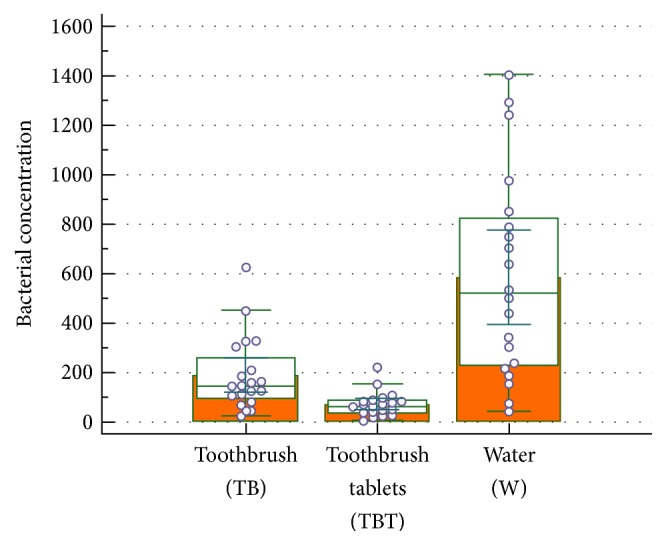
Box plot comparison between TB, TBT, and W. The graphical representation box and whiskers plot shown above, using the multiple comparison mode, is used to describe the distribution of a sample by means of simple measures of dispersion and location. The central box represents the values from the lower to upper quartile (25 to 75 percentile). The middle line represents the median. A line extends from the minimum to the maximum value, excluding “outside” and “far out” values which are displayed as separated points.

**Figure 3:**
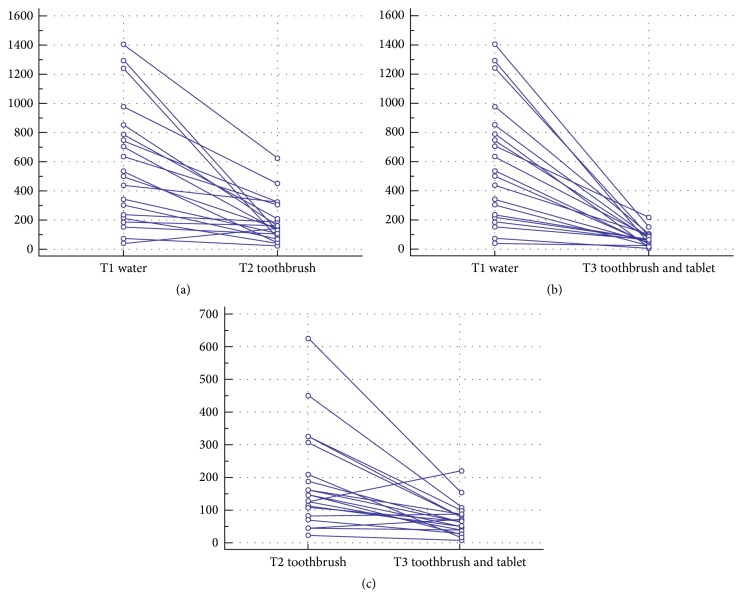
Wilcoxon test for paired data. W and TB (a), W and TBT (b), and TB and TBT (c).

All the samples were colonized by a bacterial biofilm (Table 1). The mean values of the bacterial concentration were 583 RLU, 188 RLU, and 71 RLU for the water (W), toothbrush (TB), and toothbrush and tablets (TBT), respectively (Figure 2). The median values were 518 W (95% confidence interval 248–781), 145 TB (95% confidence interval 103–205), and 64 TBT (95% confidence interval 39–85). The highest bacterial value in the TBT group was lower than the lowest value of the TB value; similarly the highest value of the TB group was lower than the lowest value of the W group. A statistical significant difference was found between the TBT group and the TB group (*p* = 0.0003) (Figure 3).

## 4. Discussion

Orthodontic treatment with clear aligners is widely accepted and used because it is a highly aesthetic and nearly invisible treatment option. A high compliance with oral hygiene procedures was found in patients treated with removable aligners, thus reducing the risk of developing plaque-related disease [10]. Several clinical [9, 13] and microbiological [8] studies showed that Invisalign appliance, even if embedded teeth and part of the keratinized gingiva nearly all day, reduces the risk of developing periodontal injury compared with fixed orthodontic appliance. This could be attributed to the fact that aligners are removable and thus allow unimpeded oral hygiene.

The fact that aligners can be removed before eating and during oral hygiene procedures does not exclude bacterial contamination and proliferation on them. Studies conducted with Scanning Electron Microscopy (SEM) highlighted the adherence of organic material and bacteria to clear aligners compromising the aesthetic aspect of them in terms of transparency. Lombardo et al. demonstrated, in vitro, using artificial saliva that the optical properties of orthodontic aligners appear to vary between brands and constituent materials but deteriorate with in vitro aging in all cases [14]. The growth of a bacterial biofilm does not only influence the aesthetic aspect of the clear aligners but also it is a potential risk factor for the development of bacteria-related disease; thus it is important to determine the most effective cleaning method. Several studies conducted on materials used in restoration procedures (such as denture materials and porcelains) showed how* S. mutans*,* C. albicans*, and streptococci accumulate on the surface of removable appliances [15, 16]. Li et al. highlighted that the nature of a surface is able to influence biofilm features such as biomass accumulation and susceptibility to antimicrobial treatments [17]. These studies showed how total biofilm mass can be reduced using daily correct hygiene procedures. A study was conducted on removable resin-made orthodontic devices and analysed the distribution frequency of* Streptococcus mutans* in the saliva of two groups of children: one group treated with resin-made removable appliances and one group untreated. A higher bacterial colonization was found in the treated group, showing how orthodontic devices may be potential carriers of bacterial infections [18]. A SEM study conducted by Diedrich on removable orthodontic appliances showed the microbiological colonization on these appliances. The results showed that using only a toothbrush was not able to provide an acceptable hygiene; on the contrary the use of ultrasound gave optimal results [19]. A recent SEM study conducted on clear aligners analysed the bacterial colonization using three different cleaning methods: running water, toothbrush and toothpaste, and toothbrush and toothpaste with sodium carbonate and sulfate [20]. This study suggested that brushing associated with the use of effervescent tablets containing sodium carbonate and sulfate is the most effective method of cleaning clear aligners. Our data are in accordance with these findings. These results can be attributable to the use of sodium carbonate and sulfate that reduce the bacterial colonization. The bioluminometer values recorded were in accordance with the results reported in the literature. Nevertheless biofilm continued to be present, even if in low concentration, in particular on the internal surfaces. This, potentially, could give rise to different problems: discoloration of the aligners, an unpleasant odour, and interaction with bacteria already present in the oral cavity. Low reported with SEM is the colonization of invisible aligners; this study described an organized growth of biofilm on the aligners' surfaces, in particular localized on more recessed and sheltered areas of the appliance, such as the cusp tips and attachment dimples [21]. Peixoto et al. performed a microbiological analysis to quantitatively evaluate the presence of* S. mutans* on the surfaces of removable orthodontic appliances. The study involved a 3-week cycle, with 1-week intervals between the weeks. During each week, three different groups of patients each followed three appliance-cleaning methods: (1) tooth brushing + baseplate brushing + sterile tap water spraying once a day; (2) tooth brushing + baseplate brushing + spraying with a 0.20% CHX-based solution on the seventh day after appliance placement; and (3) tooth brushing baseplate brushing + spraying with a 0.20% CHX-based solution on the fourth and seventh days after appliance placement. At the end of each week, the bacterial load of three randomly chosen appliances, one for each cleaning protocol, was analysed under SEM. Bacterial biofilm was detected on the surfaces of all the devices; the quantity of* S. mutans* on the surfaces treated with 0.12% CHX spray was lower than the prevalence of* S. mutans* detected on the H_2_O spray-treated surfaces and no significant difference was found between the two CHX spray protocols. It has been demonstrated and it is widely accepted that the most effective cleaning method must be still identified [22]. A study evaluated the feasibility of the removable thermoplastic appliance to adsorb hygienic solution and inhibit bacterial growth in culture and, in vivo, examined the efficacy of three hygiene protocols in reducing bacterial biofilm adherence (brushing, immersion in chlorhexidine mouthwash, and using a vibrating bath with cleaning solution). In vitro results showed the impossibility of thermoplastic appliance to adsorb substances that reduce the bacterial colonization, such as chlorhexidine. In vivo results showed that chlorhexidine and vibrating bath with cleaning solution significantly reduced baseline biofilm adherence [23]. Gracco et al. studied short-term chemical and physical changes in Invisalign appliance; morphological and structural variation occurred after their use [24]. Aligners worn for 14 days had microcracks, abraded and delaminated areas, localised calcified biofilm deposits, and loss of transparency. These alterations could induce the ecological contamination of aligners, such as for other removable orthodontic devices. A recent trial showed a similar result on the surface of Essix retainers, thus showing the bacterial colonization of removable orthodontic appliances [25]. Furthermore an analysis on the most appropriate modality of decontamination of appliances was carried out. The bacteria analysed in this study were* S. mutans* and* S. sanguis*, Actinomyces naeslundii, methicillin-resistant* Staphylococcus aureus*, and* Candida albicans*. The necessity of brushing Essix appliance, associated with the use of chemical antimicrobial method of sanitation, appears useful to reduce the bacterial count on appliances. Further studies should focus on the use of ultrasonic device for the hygienization of removable orthodontic appliances. In fact, according to some authors, the mechanical action of ultrasonic devices on dental devices may give good results even in the absence of any chemical action [26–29].

## 5. Conclusions

Within the limit of this study we can state that

- the use of sodium carbonate and sulphate effervescent tablets combined with the mechanical debridement resulted in being the most effective cleaning method;
- the bioluminometer resulted in being a reliable tool for preliminary investigation of bacterial colonization.

Further studies should investigate the use of ultrasonic devices for the cleaning of Invisalign aligners.

## Figures and Tables

**Table 1:** Value of the bioluminometer analysis; concentration value is expressed in RLU (relative light units).

Patient	T1	T2	T3
	Water	Toothbrush	Toothbrush and tablet
1	1.292	127	38
2	1.240	82	89
3	749	325	78
4	500	146	47
5	216	46	71
6	42	145	24
7	74	23	7
8	536	45	36
9	304	69	27
10	186	160	62
11	976	451	107
12	237	187	64
13	439	324	98
14	1.403	625	152
15	343	113	51
16	704	127	220
17	788	209	16
18	635	306	82
19	851	162	86
20	154	107	65
